# *Clausena Harmandiana* root extract attenuated cognitive impairments via reducing amyloid accumulation and neuroinflammation in Aβ_1-42_-induced rats

**DOI:** 10.1186/s12906-022-03591-4

**Published:** 2022-04-19

**Authors:** Nutchareeporn Nillert, Chantana Boonyarat, Jariya Umka Welbat, Komsun Bunreungthong, Ploenthip Puthongking, Wanassanun Pannangrong

**Affiliations:** 1 Department of Anatomy, Faculty of Medicine, Khon Kaen University, Khon Kaen, 40002 Thailand; 2 Faculty of Pharmaceutical Sciences, Khon Kaen University, Khon Kaen, 40002 Thailand

**Keywords:** *Clausena harmandiana*, Alzheimer’s disease, Amyloid-β, Neuroinflammation, Recognition memory

## Abstract

**Background:**

Alzheimer’s disease (AD) pathogenesis is associated with amyloid-β (Aβ)-induced neuroinflammation. In AD, the activation of microglia caused by Aβ accumulation is followed by the synthesis and release of pro-inflammatory cytokines, including interleukin-1β (IL-1β) and tumor necrosis factor-α (TNFα), and ultimately leads to cognitive impairments. *Clausena harmandiana* (CH) is a medicinal plant in the Rutaceae family and has been used in folk medicine to relieve illnesses such as stomachache and headache, and as a health tonic. Interestingly, CH root extract (CHRE) has several anti-inflammatory and other pharmacological activities, but there are no studies in AD-like animal models.

**Objectives:**

This study aims to evaluate the effects of CHRE on cognitive impairments, increased Aβ_1–42_ protein levels, and neuroinflammation in Aβ_1–42_-induced rats.

**Methods:**

Forty-eight adult male Sprague-Dawley rats (250–300 g) were randomly divided into 6 groups (*n* = 8) of the sham control, V + Aβ, CB + Aβ CHRE125 + Aβ, CHRE250 + Aβ, and CHRE500 + Aβ. Sodium carboxymethylcellulose, Celebrex (10 mg/kg BW) and CHRE (125, 250, and 500 mg/kg BW) were given orally or without any treatment for 35 days. On day 21, aggregated Aβ_1–42_ at a concentration of 1 μg/μl were injected into both lateral ventricles (1 μl/side) of all treated rats, while sterilized normal saline were injected to untreated rats. Ten days later, the novel object recognition test was performed to assess their recognition memory. At the end of the test period, an overdose of thiopental sodium (120 mg/kg BW) and transcardial perfusion with 0.9% normal saline solution were used to euthanize all rats. Then Aβ_1–42_ protein levels and the expression of inflammatory markers (CD11b-positive microglia, IL-1β, and TNFα) were investigated in the cerebral cortex and hippocampus.

**Results:**

Pretreatment with CHRE at all doses could attenuate short- and long-term impairments in recognition memory. Additionally, CHRE also inhibited the increase of Aβ_1–42_ protein levels and the expression of inflammatory markers in both brain regions as well as receiving Celebrex.

**Conclusions:**

This suggests that preventive treatment of CHRE might be a potential therapy against cognitive impairments via reducing Aβ_1–42_ protein levels and neuroinflammation caused by Aβ_1–42_.

**Supplementary Information:**

The online version contains supplementary material available at 10.1186/s12906-022-03591-4.

## Background

Alzheimer’s disease (AD) is an irreversible neurodegenerative disease and the most common type of dementia in the elderly. It is characterized by progressive cognitive impirments combined with memory loss. Two neuropathological hallmarks are neurofibrillary tangles of the tau protein and senile plaques (SP) [1, 2]. Amyloid-β 1–42 (Aβ_1–42_) is the main component of SP that is the most significant toxic fragment to various brain regions such as the cerebral cortex and hippocampus [3–6]. Abnormal Aβ accumulation causes neurotoxicity through various mechanisms, particularly neuroinflammation, which is an innate immune response in the central nervous system (CNS) against various pathological triggers including neuronal death or proteins aggregation [7]. In AD, Aβ-induced neuroinflammation occurs through the binding of Aβ to receptors expressed on microglia, particularly clusters of differentiation molecule 11b (CD11b) that play an important role in Aβ clearance [8]. Chronic microglial activation can also trigger the nuclear factor-κB (NF-κB) signaling pathway, and the subsequent synthesis of pro-inflammatory cytokines, especially interleukin-1β (IL-1β) and tumor necrosis factor-α (TNFα) [9, 10]. It was found that the brain tissue and cerebrospinal fluid of AD patients presented increased IL-1β and TNFα levels [11], inhibiting microglial phagocytosis, which in turn intensifies Aβ accumulation and neuroinflammation [12, 13]. Chronic expression of TNFα and IL-1β also increased neuronal death [14] and enhances the synthesis of other pro-inflammatory cytokines [15], resulting in inhibition of long-term potentiation and impaired learning and memory [16]. Recognition memory is a type of memory that is often impaired in patients affected by neurodegenerative diseases or brain injuries [17] as well as in Aβ-induced rats [18]. Several studies reported that inhibiting inflammation in AD can slow cognitive decline [19, 20]. Non-steroidal anti-inflammatory drugs (NSAIDs) are commonly used to prevent and delay the onset of AD [21, 22]. Celecoxib (Celebrex) belongs to the class of NSAIDs called selective cyclooxygenase-2 (COX-2) inhibitors, which are highly safe and less toxic than other NSAIDs [23, 24]. Celecoxib exhibits anti-inflammatory and neuroprotective effects by decreasing microglial activation and pro-inflammatory cytokine expression in the hippocampus of Aβ-induced rats [20]. Nevertheless, prolonged exposure to NSAIDs can cause various side effects such as gastrointestinal bleeding and ulcer, arterial wall damage, and nephrotoxicity [25–28]. Researchers have recently shown a growing interest in various natural products with anti-inflammatory due to few adverse effects. *Clausena harmandiana* (CH; song fa dong in Thai) is a herb of the Rutaceae family that has been used as a folk medicine for the treatment of illness, stomachache, headache, and herbal health tonic [29, 30]. Nordentatin is a type of coumarin compound obtained from a natural product, it is a major active ingredient isolated from the root bark of CH (CHR) [31]. According to a previous study, 100 g of CHR extract (CHRE) contained 0.532 g of nordentatin [32]. The pharmacological activities of this compound included antioxidant activity [32], promoted neurite outgrowth [32], and improved Aβ-induced cognitive impairment [33]. Interestingly, there have been no published reports of serious toxicity from CHRE in either in vitro or in vivo models [32, 34]. However, its anti-neuroinflammatory effects and potential ability to alleviate cognitive impairment in animal models of AD have not been investigated.

Thus, this study aims to examine the effects of CHRE on Aβ_1–42_-induced cognitive impairments, Aβ_1–42_ protein levels and neuroinflammation in rats.

## Methods

### Plant material and preparation of CHRE

The root bark of CH (CHR) was collected from Roi Et province, Thailand. The plant was identified voucher specimen (KKU No. 21145; Teerapat Bootchan 67) and deposited at Faculty of Sciences, Khon Kaen Univerisity, Khon Kaen, Thailand. CHRE was prepared by Faculty of Pharmaceutical Sciences, Khon Kaen University, Khon Kaen, Thailand, and permitted to be used in this study. Air-dried CHR was macerated with 70% ethanol and periodically stirred at room temperature for 3 days. The extract was then filtered and concentrated using a rotary evaporator at 40 °C and stored in a refrigerator at 2–8 °C until use. The extracted yield of CHRE was 19.7% of the wet weight [34].

### Animal treatment

**Fig. 1:**
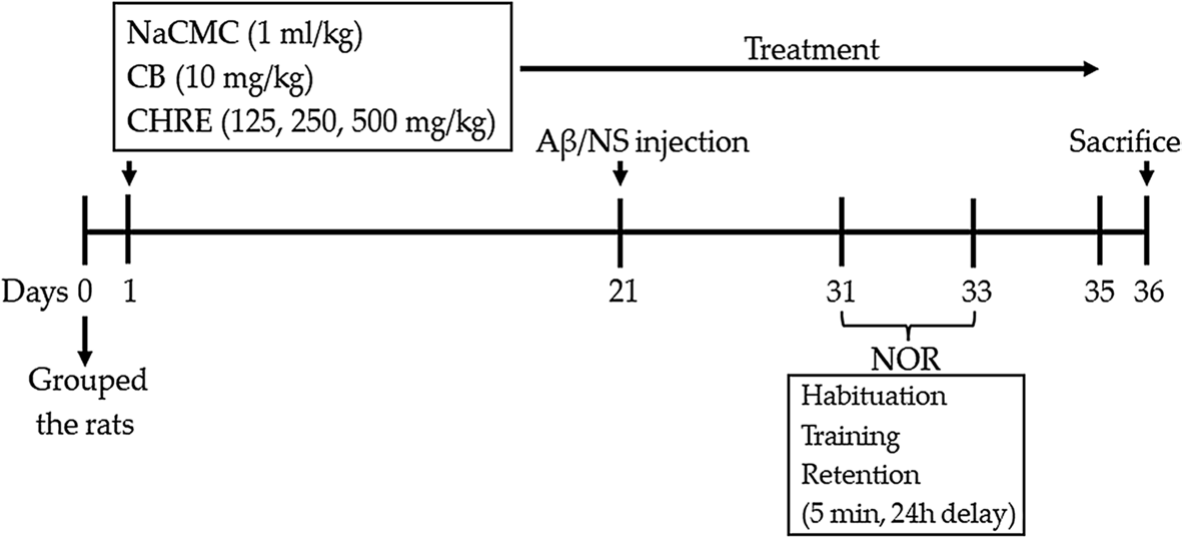
Schematic diagram of drug treatment and behavioral tests. Rats were injected with Aβ_1–42_ into the lateral ventricle on each side after 21 days of drug treatment. NaCMC: sodium carboxymethylcellulose; CB: Celebrex; CHRE: *Clausena harmandiana* root extract; Aβ: amyloid-β; NS: normal saline; NOR: novel object recognition

Forty-eight healthy adult male Sprague-Dawley rats (250–300 g) were obtained from Nomura Siam International Co., Ltd. (Bangkok, Thailand). The rats were housed in the Northeast Laboratory Animal Center, Khon Kaen University at room temperature (23 ± 2 °C) under a 12-h light/dark cycle (lights on from 06:00 to 18:00) with free access to food and water. All studies were carried out following the procedures laid out in the guide for the care and use of laboratory animals under the supervision of the Northeast Laboratory Animal Center, Khon Kaen University, Khon Kaen, Thailand. The experimental design was approved by the Institutional Animal Care and Use Committee of Khon Kaen University (Approval No. IACUC-KKU-25/61; Suppl. Fig. 1). After 1 week of acclimatization, the rats were randomly divided into 6 groups (*n* = 8/group). Group 1 was a sham control (SC) group, rats were injected with sterilized normal saline (NS). Group 2 was a vehicle plus amyloid-β (V + Aβ) group, rats were received 0.5% sodium carboxymethylcellulose (NaCMC) and induced memory impairments with Aβ_1–42_ injection. Group 3 was a Celebrex plus amyloid-β (CB + Aβ; a positive control) group, rats were received Celebrex at 10 mg/kg body weight (BW) and induced memory impairments with Aβ_1–42_ injection. Groups 4, 5, and 6 were the CHRE125 plus amyloid-β (CHRE125 + Aβ), CHRE250 plus amyloid-β (CHRE250 + Aβ) and CHRE500 plus amyloid-β (CHRE500 + Aβ), rats were received CHRE at 125, 250, and 500 mg/kg BW, respectively and induced memory impairments with Aβ_1–42_ injection. NaCMC, Celebrex, and CHRE were orally administered once daily for 35 consecutive days. On day 21, the rats in groups 2–6 were injected with 1 μl of aggregated Aβ_1–42_ peptide into the lateral ventricle on each side, whereas group 1 was injected with the same dose of sterilized NS. Ten days after Aβ_1–42_ injection, the rats were tested for learning and memory using a novel object recognition (NOR) test (Fig. 1). At the end of the experiment, the rats were euthanized by an anesthesia overdose (120 mg/kg BW of thiopental sodium; Jagsonpal Pharmaceuticals Ltd., India) and transcardial perfusion with 0.9% NS solution, and then Aβ_1–42_ protein levels and the expression of CD11b-positive microglia, IL-1β, and TNFα in the cerebral cortex and hippocampus were investigated by immunohistochemistry or western blot analysis.

### Aβ_1–42_ injection

Amyloid-β peptide 1–42 (Aβ_1–42_; Enzo Life Sciences, Farmingdale, NY, USA) was dissolved in 5% acetic acid at a concentration of 1 μg/μl, and the solution was incubated at 37 °C for 24 h to induce peptide aggregation [35]. The rats were anesthetized with thiopental sodium (Jagsonpal Pharmaceuticals Ltd., India; 80 mg/kg BW, intraperitoneal), followed by a single injection of aggregated Aβ_1–42_ peptide or sterilized NS into the lateral ventricles bilaterally (1 μl/side) at a rate of 0.2 μl/min [36, 37] using the following coordinates: AP − 0.8 mm from bregma, ML ± 1.5 mm from bregma, and SI − 3.8 mm from dura mater [18, 38]. After injection, the rats were placed on a warm pad (32–33 °C) until they awoke and were returned to their cages [39].

### Novel object recognition test

The novel object recognition (NOR) test was performed in an open field arena (50 cm × 50 cm × 40 cm) in a quiet environment. The test consisted of three phases: habituation, training, and retention. In the habituation phase, each rat was allowed to freely survey the empty arena for 5 min. During the training phase, each rat was allowed 5 min to explore two identical objects that had been placed in the arena at different locations. The retention phase was divided into 2 sub-periods with a 5 min and 24 h delay to assess short- and long-term memory, respectively. Five minutes or 24 h after training, one of the objects was replaced with a novel object, and each rat was again placed in the arena to explore the objects for 5 min. After each rat finished the test, the arena was cleaned with 70% ethanol to remove any odor. Exploration time was recorded using Noldus EthoVision XT version 12 when the rat’s mouth, nose, or paw was less than 2 cm from an object. The ability to recognize the novel object was expressed as a discrimination index (DI): the difference between the amount of time spent exploring the novel and familiar object divided by total exploration time (DI = TN - TF/TN + TF). The DI can range from − 1 to 1, with positive and negative scores indicating more time spent with the novel and familiar object, respectively, and a zero indicating a null preference [18].

### Tissue processing

After finishing treatment, all rats were deeply anesthetized with an overdose (120 mg/kg BW) of thiopental sodium (Jagsonpal Pharmaceuticals Ltd., India) and transcardially perfused with 0.9% NS solution. Then, the brains were quickly removed and separated into left and right hemispheres. The left hemisphere was cryoprotected in 30% sucrose solution and submerged in ice-cold 4% paraformaldehyde solution for immunohistochemical investigation using a free-floating technique. The cerebral cortex and hippocampus from the right hemisphere were isolated and stored at − 80 °C for Western blot analysis.

### Immunohistochemistry

The frozen brains were cut into serial coronal sections at 35 μm thickness using a cryostat and then were washed with 0.1 M tris-buffered saline (TBS) 3 times for 5 min. Free-floating sections were incubated with 0.3% hydrogen peroxide (H_2_O_2_) for 15 min to suppress the endogenous peroxidase activity and again washed with TBS. The sections were blocked with 1% bovine serum albumin (BSA; Merck Millipore, Germany) at room temperature for 1 h and incubated with mouse monoclonal anti-integrin αM (CD11b) primary antibody (1:100; Merck Millipore, Germany) at 4 °C overnight. After washing with TBS, the sections were incubated with horseradish peroxidase (HRP) conjugated goat anti-mouse IgG secondary antibody (1:500; Invitrogen, Carlsbad, CA, USA) at room temperature for 2 h. The sections were washed with TBS and incubated with 0.001% of diaminobenzidine tetrahydro-chloride dihydrate (DAB; Sigma Aldrich, USA) containing 0.003% H_2_O_2_ for 20 min. Then, the sections were mounted on gelatin-coated glass slides, dehydrated, and cover-slipped with DPX (Sigma, St. Louis, MO, USA). The immunostained sections were viewed under a light microscope (Nikon ECLIPSE E200 MVR microscope) at X400 magnification and assessed using ImageJ software (Windows version, National Institutes of Health, Bethesda, MD, USA). Six digitized images of the cerebral cortex and hippocampus were obtained from 1 image per area of the frontal cortex (FC), parietal cortex area 2 (PC2) or temporal cortex area 1 (TC1), perirhinal cortex (PRC), and piriform cortex (PiC), as well as 2 images per area of parietal cortex area 1 (PC1), cornu ammonis area 1 (CA1), cornu ammonis area 2/3 (CA2/3), and dentate gyrus (DG). Six sections were then selected from each rat for analysis. Results were represented as a percentage of immunoreactive area difference from control, which was calculated using the following formula: immunoreactive area = [areas of CD11b-positive microglia (positive pixels)/total area of the image (total pixels)] [18, 40].

### Western blot analysis

Cerebral cortex or hippocampus tissue was homogenized separately using grinder with the cold lysis buffer (pH 7.6) containing 20 mM tris base (Sigma-Aldrich, USA), 1 mM ethylene glycol tetraacetic acid (Sigma-Aldrich, USA), 320 mM sucrose (Loba Chemical Pvt. Ltd., India), 0.1% triton X 100 (Sigma-Aldrich, USA), 1 mM sodium fluoride (NaF), 10 mM β-glycerophosphate disodium salt hydrate (Sigma-Aldrich, USA) and Sigma*FAST* protease inhibitor cocktail (Sigma Aldrich, USA) to extract total proteins. The homogenized samples were centrifuged at 13,000 rpm at 4 °C for 10 min. The protein concentration was determined using a NanoDrop (NanoDrop ND-1000 Spectrophotometer V3.5 User’s Manual, NanoDrop Technologies, USA). The samples (100 μg) were heat-denatured at 95 °C for 5 min, separated with 12% sodium dodecyl sulfate-polyacrylamide gel electrophoresis (SDS-PAGE; Bio-Rad Laboratories GmbH, Munich, Germany), and subsequently transferred onto the nitrocellulose membrane (Bio-Rad Laboratories GmbH, Munich, Germany). The membrane was blocked with 5% BSA (Merck Millipore, Germany) in 0.1% tris-buffer saline containing tween 20 (TBST) at room temperature for 1 h and then probed with rabbit polyclonal anti-Aβ_1–42_ (1:2000; Abcam, UK), mouse monoclonal anti-IL-1β (1:300; Santa Cruz Biotechnology, USA), mouse monoclonal anti-TNF-α (1:300; Santa Cruz Biotechnology, USA), and mouse monoclonal anti-glyceraldehyde 3 phosphate dehydrogenase (GAPDH; 1:20,000; Abcam, UK) primary antibodies in TBST at 4 °C for overnight. After washing with TBST, the membrane was incubated with peroxidase conjugated goat anti-rabbit IgG (1:5000; Merck Millipore, Germany) or HRP conjugated goat anti-mouse IgG secondary antibodies (1:2000; Thermo Fisher Scientific, USA) at room temperature for 2 h. The protein bands were visualized with enhanced chemiluminescence (ECL) detection reagents (Thermo Fisher Scientific, USA) and a gel imaging system (Image Quant 400, GH Healthcare, USA), and analyzed using ImageJ software (Windows version, National Institutes of Health, USA). GAPDH was used as a loading control, and the results were represented as the percentage difference from control.

### Statistical analysis

All data were presented as mean ± standard error of the mean (SEM) and analyzed using SPSS 23.0 software. Statistical analysis was performed using a one-way analysis of variance (ANOVA) followed by a Tukey post-hoc test for multiple comparisons. A *P* value < 0.05 was considered statistically significant.

## Results

### Effects of CHRE on cognitive impairment in Aβ_1–42_-injected rats

**Fig. 2:**
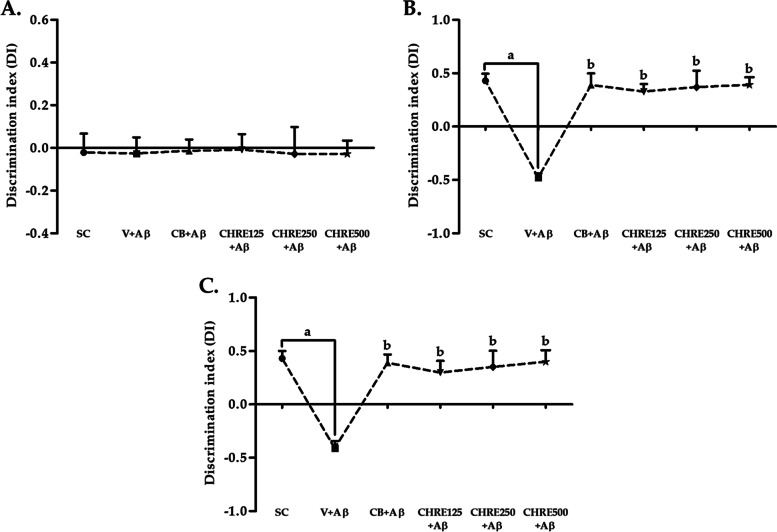
Effects of CHRE on cognitive impairment in Aβ_1–42_-injected rats in the novel object recognition (NOR) test. The discrimination index (DI) in the training phase (**A**), after a 5 min delay (**B**) and a 24 h delay (**C**) of the NOR test. Data are expressed as mean ± SEM; a = significant difference from the SC group at *P* < 0.001, and b = significant difference from the V + Aβ group at *P* < 0.001

Cognitive impairments, such as impaired object recognition, are typically found in AD patients and animal models. The NOR test was performed on days 31 to 33 to investigate whether CHRE ameliorated such cognitive impairment in Aβ_1–42_ injected rats (Fig. 2). During the training phase, there was no marked difference in the time spent exploring the two identical objects in any group (Fig. 2A). In the retention phase, only the V + Aβ group displayed obvious cognitive impairments, indicated by significant decreases in both short- and long-term DIs compared with the SC group (Fig. 2B-C). These cognitive impairments (both short- and long-term) were ameliorated by administration of CHRE, as demonstrated by the significantly higher DIs in the CHRE125 + Aβ, CHRE250 + Aβ, and CHRE500 + Aβ groups (similar to the CB + Aβ group) compared with the V + Aβ group (Fig. 2B-C). This shows that CHRE improved object recognition impairment in Aβ_1–42_ injected rats. However, there was no significant difference in locomotor activity (velocity of movement or total distance traveled) among groups (Suppl. Fig. 2).

### Effects of CHRE on Aβ_1–42_ protein levels in the cerebral cortex and hippocampus of Aβ_1–42_ injected rats

**Fig. 3:**
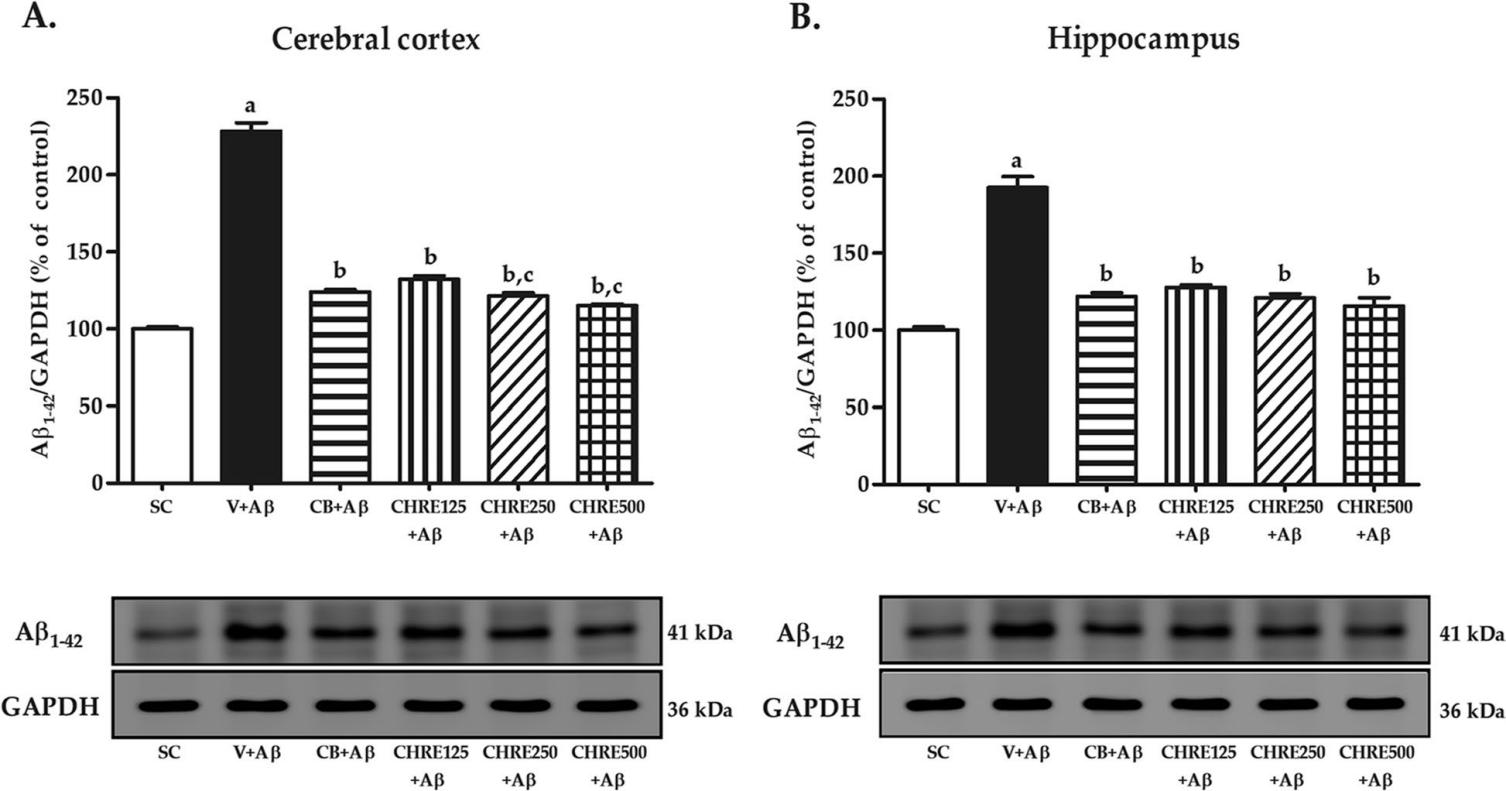
Effects of CHRE on Aβ_1–42_ protein levels in the brain of Aβ_1–42_ injected rats. Representative Western blot imaging and quantitative analysis of Aβ_1–42_ protein levels in the cerebral cortex (**A**) and hippocampus (**B**) using Western blot. GAPDH is used as an internal control. Data are expressed as mean ± SEM; a = significant difference from the SC group at *P* < 0.001, b = significant difference from the V + Aβ group at *P* < 0.001, and c = significant difference from the CHRE125 + Aβ group at *P* < 0.001. The original uncropped Western blots for Aβ_1–42_ protein and GAPDH were represented in Suppl. Fig. 3

Increases in brain Aβ protein levels are the main contributing factor to AD progression. Western blot was performed to determine whether CHRE decreased Aβ_1–42_ protein levels in the rat brain (Fig. 3). Injection of aggregated Aβ into both lateral ventricles significantly increased Aβ_1–42_ protein levels in the cerebral cortex and hippocampus of the V + Aβ group compared with the SC group (Fig. 3A-B). However, administration of Celebrex and CHRE significantly decreased Aβ_1–42_ protein levels in both brain regions of rats in the CB + Aβ, CHRE125 + Aβ, CHRE250 + Aβ, and CHRE500 + Aβ groups compared with the V + Aβ group (Fig. 3A-B), particularly CHRE250 and CHRE500 (Fig. 3A).

### Effects of CHRE on the expression of CD11b-positive microglia in the cerebral cortex and hippocampus of Aβ_1–42_-injected rats

**Fig. 4:**
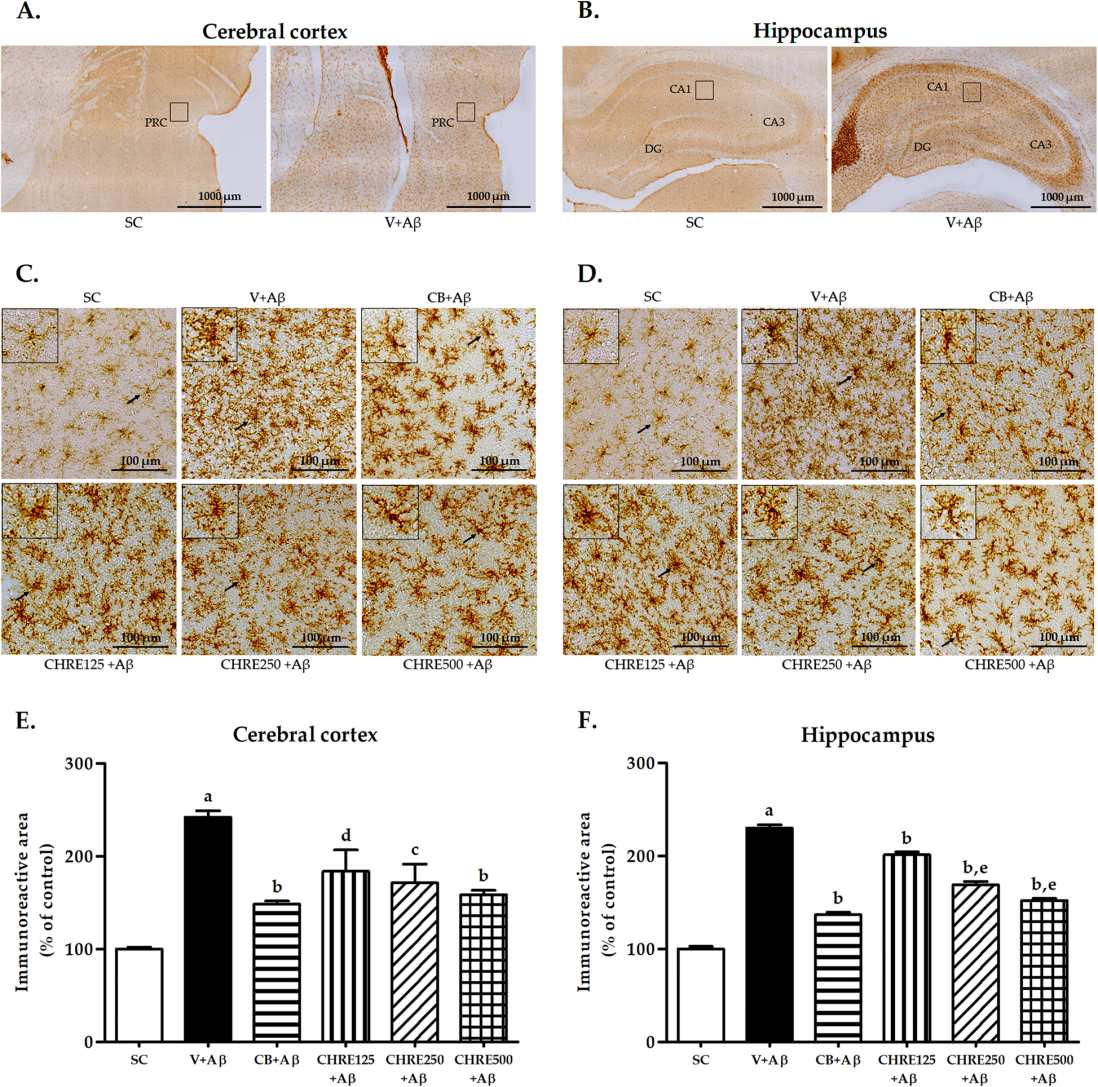
Effects of CHRE on the expression of CD11b-positive microglia in the brain of Aβ_1–42_-injected rats. Representative photomicrographs of CD11b-positive microglia in the cerebral cortex (**A**) and hippocampus (**B**) of SC and Aβ-induced rats at low magnification. Representative photomicrographs of CD11b-positive microglia in the cerebral cortex (**C**) and hippocampus (**D**) of SC and Aβ_1–42_-injected rats at high magnification. The density of CD11b-positive microglia in the cerebral cortex (**E**) and hippocampus (**F**) measured using immunohistochemistry. Data are expressed as mean ± SEM; a = significant difference from the SC group at *P* < 0.001, b, c, and d = significant difference from the V + Aβ group at *P* < 0.001, *P* < 0.01, and *P* < 0.05, respectively, and e = significant difference from the CHRE125 + Aβ group at *P* < 0.001

Increased expression of microglial markers, such as CD11b, has been widely reported in AD. IHC was performed to evaluate whether CHRE reduced the expression of CD11b-positive microglia in the rat brain (Fig. 4). Fig. 4A-B shows photomicrographs of CD11b-positive microglia in the cerebral cortex and hippocampus of SC and Aβ-induced rats at low magnification. In general, microglia in the cerebral cortex and hippocampus were ramified (small cell bodies, thin and long branches; Fig. 4C-D), and the expression of CD11b-positive microglia was lower than in the SC group (Fig. 4E-F). Aβ_1–42_ injection caused morphological changes to the microglia, which took on an amoeboid form (large cell bodies, thick and short branches) as found in the V + Aβ group (Fig. 4C-D). Moreover, Aβ_1–42_ injection significantly elevated the expression of CD11b-positive microglia in the cerebral cortex and hippocampus of the V + Aβ group compared with the SC group (Fig. 4E-F). However, administration of CHRE significantly reduced the expression of CD11b-positive microglia in both brain regions of rats in the CHRE125 + Aβ, CHRE250 + Aβ, and CHRE500 + Aβ groups (to levels similar to those in the CB + Aβ group) compared with the V + Aβ group (Fig. 4E-F). Some amoeboid microglia were found in all treatment groups (Fig. 4C-D). Interestingly, CHRE250 and CHRE500 decreased the expression of CD11b-positive microglia in the hippocampus to a greater extent than CHRE125 (Fig. 4F).

### Effects of CHRE on the expression of IL-1β and TNFα in the cerebral cortex and hippocampus of Aβ_1–42_-injected rats

**Fig. 5:**
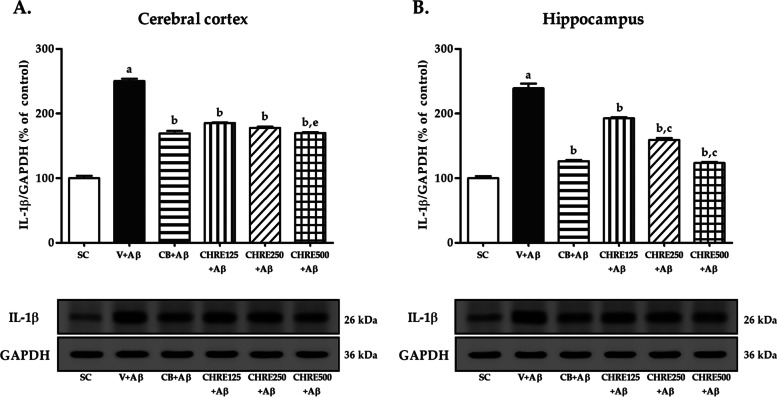
Effects of CHRE on the expression of IL-1β in the brain of Aβ_1–42_-injected rats. Representative western blot imaging and quantitative analysis of IL-1β in the cerebral cortex (**A**) and hippocampus (**B**) using Western blot. GAPDH is used as an internal control. Data are expressed as mean ± SEM, a = significant difference from the SC group at *P* < 0.001, b = significant difference from the V + Aβ group at *P* < 0.001, and c and e = significant difference from the CHRE125 + Aβ group at *P* < 0.001 and *P* < 0.05, respectively. The original uncropped Western blots for IL-1β and GAPDH were represented in Suppl. Fig. 4

**Fig. 6:**
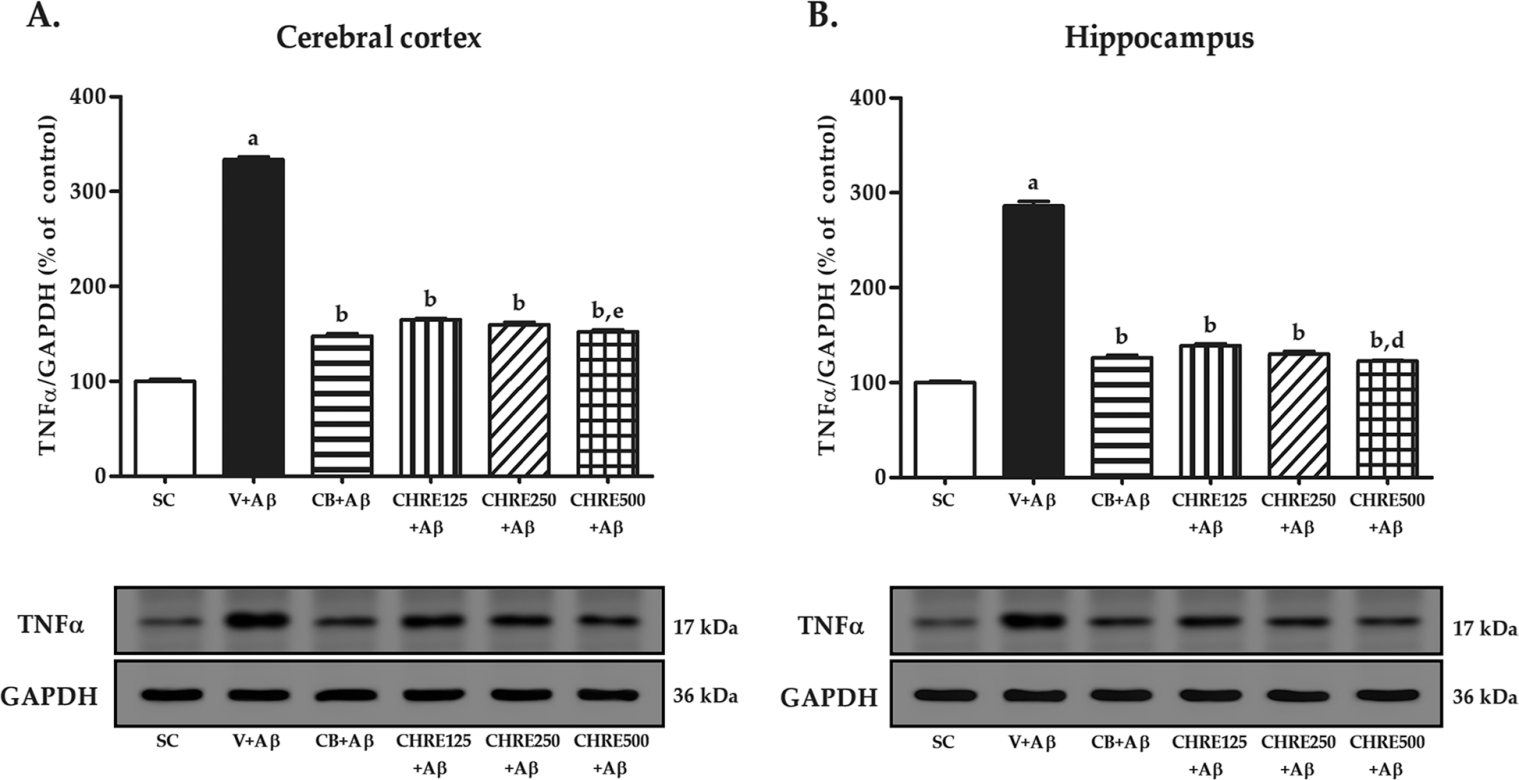
Effects of CHRE on the expression of TNFα in the brain of Aβ_1–42_-injected rats. Representative western blot imaging and quantitative analysis of TNFα in the cerebral cortex (**A**) and hippocampus (**B**) using Western blot. GAPDH is used as an internal control. Data are expressed as mean ± SEM, a = significant difference from the SC group at *P* < 0.001, b = significant difference from the V + Aβ group at *P* < 0.001, and d and e = significant difference from the CHRE125 + Aβ group at *P* < 0.01 and *P* < 0.05, respectively. The original uncropped Western blots for TNFα and GAPDH were represented in Suppl. Fig. 5

The increment of major pro-inflammatory cytokines, including IL-1β and TNFα, plays a crucial role in neuroinflammation and cognitive impairment in AD. Western blotting was performed to assess whether CHRE attenuated the expression of IL-1β (Fig. 5) and TNFα (Fig. 6) in the rat brain. The results showed that Aβ_1–42_ injection dramatically enhanced the expression of IL-1β and TNFα in the cerebral cortex and hippocampus of rats in the V + Aβ group compared with those in the SC group (Fig. 5A-B and 6A-B). However, administration of Celebrex and CHRE significantly attenuated the expression of IL-1β and TNFα in both brain regions of rats in the CB + Aβ, CHRE125 + Aβ, CHRE250 + Aβ, and CHRE500 + Aβ groups compared with those in the V + Aβ group (Fig. 5A-B and 6A-B). Importantly, CHRE500 reduced the overall expression of both cytokines to a greater extent than CHRE125 (Fig. 5A-B and 6A-B), while only CHRE250 resulted in a greater reduction of IL-1β in the hippocampus (Fig. 5B).

## Discussion

The focus of our study was to assess the pharmacological activities of CHRE in animal models of inflammation hypothesis of AD. We found that CHRE improved cognitive impairments, and decreased Aβ_1–42_ protein levels and inflammatory markers in Aβ_1–42_-induced rats. Various animal models were used to mimic pathologies occurring similar to those seen in AD patients, such as cognitive impairments, Aβ accumulation, and neuroinflammation [41–43]. Aβ_1–42_ is another model used to inject into the ventricles of the animal brain. This causes memory impairment and neuroinflammation [18]. Therefore, we applied this model to our study. The results of the retention phase of NOR showed Aβ_1–42_ injection impaired both short- and long-term recognition memory, indicating the successful establishment of an amnesia model. Pretreatment with CHRE at all dosages (125, 250, and 500 mg/kg BW) markedly improved Aβ_1–42_-induced impairment of both short- and long-term memory. This is consistent with a previous report demonstrating that nordentatin at a dose of 50 μmol/kg BW ameliorated cognitive impairments caused by Aβ injection as assessed using the Y-maze test [33].

Moreover, we found that Aβ_1–42_ injection increased Aβ accumulation by increasing the level of Aβ_1–42_ proteins in the cerebral cortex and hippocampus. However, pretreatment with CHRE at all doses reduced these effects. This result is supported by a previous study, which found that 7-hydroxy-5-methoxy-4-methyl-3-(4-methyl piperazin-1-yl)-coumarin (IMM-H004) inhibited Aβ accumulation in the hippocampus [44]. Another study also found that coumarins may interfere with the process of Aβ accumulation by interacting with aromatic residues within the hydrophobic core of Aβ [45]. These compounds also reduce the release of Aβ from amyloid precursor protein (APP) via inhibition of β-secretase activity [46]. These properties may have been associated with the improvements we observed in Aβ_1–42_ protein levels. Excessive microglial activation is a predominant feature of chronic neuroinflammation. In AD, Aβ accumulation can activate the surrounding microglia to facilitate its clearance [47]. It is still unclear whether microglia lose their efficacy or even become detrimental in the later stages of AD. A major challenge is to identify the specific pathways and molecular mechanisms that could be early indicators of brain damage and protect the brain from degeneration. The evidence suggests that disease-associated microglia (DAM), a recently identified subset of CNS resident macrophages found at sites of neurodegeneration [48], might play such a protective role. Recent studies have shown that DAM has a specific sensory mechanism, which includes the triggering receptors expressed on the myeloid cell 2 (TREM2) signaling pathway [49, 50], to detect damage within the CNS in the form of neurodegeneration-associated molecular patterns (NAMPs). Transcriptional analysis of DAM and human genome-wide association studies (GWASs) have examined the potential function of DAM pathways in the neurodegenerative brain [48, 51, 52]. Therefore, manipulating DAM may create new therapeutic opportunities for AD. However, the technical limitations involved in analyzing heterogeneous microglia populations make it difficult to accurately determine the immune cell types, the status of the relevant immune cells, and the states involved in brain disease precisely. While some studies have focused on microglial activation through basal receptors including CD11b.

This study showed that Aβ_1–42_ injection enhanced the expression of CD11b-positive microglia in the cerebral cortex and hippocampus. This is consistent with the above-mentioned results showing that increased Aβ_1–42_ protein levels promote microglial phagocytosis through activation of their receptors. Additionally, IL-1β and TNFα expressions also increased in both brain regions of the Aβ_1–42_-injected rats, these effects were reversed via pretreatment with CHRE at all dosages. Similarly, a previous study reported that several coumarins also have anti-inflammatory effects. For example, auraptene was able to inhibit microglial activation and COX-2 expression from astrocytes and neuronal cell death in the hippocampus [41]. Besides, xanthotoxol, IMM-H004, and osthole have been shown to reduce IL-1β, IL-6, TNFα, and nitric oxide (NO) in vivo [53–55]. The ability of coumarins to relieve neuroinflammation may involve interference with the binding of Aβ and microglia receptors [56] that induced an inhibitory effect on the nuclear translocation of NF-κB, the phosphorylation of Jun N-terminal kinase (JNK), and p38 mitogen-activated protein kinase (MAPK) pathways, leading to suppression of the synthesis and release of pro-inflammatory cytokines [45, 57]. In addition, coumarins exhibit acetylcholinesterase (AChE) inhibitory activity, which in turn increases acetylcholine (ACh) levels in the brain [58–60], ultimately leading to decreases in cognitive impairment caused by Aβ_1–42_ injection. Inflammatory markers were also found to decrease in both brain regions in Celebrex-treated rats. This is consistent with previous studies, which reported reverse in COX-2, IL-1α, IL-1β, IL-6 and IL-12 and BDNF in soluble amyloid-β (sAβ)-treated rats that received Celecoxib [20]. Hence, this drug is commonly used as a positive control for studies in rat AD models. The dosing regimen could be critical, as using NSAIDs intermittently versus constant exposure could modulate the immune system differently. Both beneficial and detrimental effects of microglia activation have been described and depend on factors such as age, disease stage, etc. [61, 62]. In the early preclinical stage of AD, mild microglial activation could play a beneficial role. As with NSAIDs, the role of CHRE in anti-inflammatory effects is being studied. Interestingly, medium or high doses of CHRE were more effective than the lower doses in improving Aβ_1–42_ protein levels and neuroinflammation. The reason for this was that all dosages were within the therapeutic range [63], meaning that the CHRE at higher concentrations was able to interact with the aromatic residues of Aβ and interfere with the binding of Aβ and microglial receptors more effectively than at lower concentrations. The limitation of this study is the pharmacokinetics of CHRE have not been reported in any publication, only the coumarins have been published. It has shown that after oral administration coumarins are immediately absorbed via the mucosa of the gastrointestinal tract and then disseminated throughout the body. Since they are mainly metabolized in the liver by the first-pass effect, only 2–6% were found intact in the systemic circulation [64, 65]. In addition, they rapidly distribute into the brain which can be detected at 15 min after administration, and their contents are closely related to plasma concentrations [66]. However, the pharmacokinetics of CHRE may be reported in the future.

All of this shows that the increment of Aβ and pathological changes that occur in the brain due to neuroinflammation relate to cognitive impairments and the progression of AD. Consequently, reductions of Aβ and neuroinflammation may ameliorate cognitive impairments and delay the progression of AD. We found that administration of CHRE for 35 consecutive days mitigated both short- and long-term recognition memory impairment caused by the Aβ_1–42_ injection. It also decreased Aβ_1–42_ protein levels and neuroinflammation in the cerebral cortex and hippocampus, both of which are involved in recognition memory [39, 40]. This indicates that the preventive action of CHRE reduced Aβ accumulation, neuroinflammation, and cognitive impairments caused by Aβ_1–42_. So CHRE is an attractive option in the investigation, analysis, and development of pharmacological agents that have the potential to alleviate impairments in AD.

## Conclusions

This study showed that CHRE improved cognitive impairments in Aβ_1–42_-induced rats through decreased Aβ_1–42_ protein levels and neuroinflammation, particularly the expressions of CD11b-positive microglia, IL-1β, and TNFα in the cerebral cortex and hippocampus.

## Supplementary Information

**Additional file 1:** Suppl. Fig. 1

**Additional file 2:** Suppl. Fig. 2

**Additional file 3:** Suppl. Fig. 3

**Additional file 4:** Suppl. Fig. 4

**Additional file 5:** Suppl. Fig. 5

## Data Availability

All data generated or analyzed during this study are included in this published article and its supplementary information files.

## Abbreviations

ACh: Acetylcholine
AChE: Acetylcholinesterase
AD: Alzheimer’s disease
ANOVA: Analysis of variance
AP: Anterior-posterior
APP: Amyloid precursor protein
Aβ: Amyloid-β
BDNF: Brain-derived neurotrophic factor
BSA: Bovine serum albumin
BW: Body weight
°C: Degrees Celsius
CA: California
CA1: Cornu ammonis area 1
CA2/3: Cornu ammonis area 2/3
CB: Celebrex
CD11b: Clusters of differentiation molecule 11b
CH: *Clausena harmandiana*
CHR: *Clausena harmandiana* root
CHRE: *Clausena harmandiana* root extract
cm: centimeter
CNS: Central nervous system
COX-2: Cyclooxygenase-2
DAB: Diaminobenzidine tetrahydro-chloride dihydrate
DAM: Disease-associated microglia
DG: Dentate gyrus
DI: Discrimination index
DPX: Dibutylphthalate polystyrene xylene
etc.: et cetera
ECL: Enhanced chemiluminescence
FC: Frontal cortex
Fig: Figure
g: gram
GAPDH: Glyceraldehyde 3 phosphate dehydrogenase
GWASs: Gsenome-wide association studies
h: hour
H_2_O_2_: Hydrogen peroxide
HRP: Horseradish peroxidase
IgG: Immunoglobulin G
IHC: Immunohistochemistry
IL-1β: Interleukin-1β
IMM-H004: 7-hydroxy-5-methoxy-4-methyl-3-(4-methyl piperazin-1-yl)-coumarin
JNK: Jun N-terminal kinase
kg: kilogram
Ltd.: Limited
μg: microgram
μl: microliter
μm: micrometer
M: Molar
MAPK: Mitogen-activated protein kinase
MD: Maryland
mg: milligram
min: minute
ML: Medial-lateral
mM: millimolar
MO: Missouri
NaCMC: sodium carboxymethylcellulose
NaF: sodium fluoride
NAMPs: Neurodegeneration-associated molecular patterns
NF-κB: Nuclear factor kappa-light-chain-enhancer of activated B cells
NO: Nitric oxide
NOR: Novel object recognition
NS: Normal saline
NSAIDs: Non-steroidal anti-inflammatory drugs
NY: New York
PC1: Parietal cortex area 1
PC2: Parietal cortex area 2
pH: positive potential of the Hydrogen ions
PiC: Piriform cortex
PRC: Perirhinal cortex
Pvt: Private
sAβ: soluble amyloid-β
SC: Sham control
SEM: Standard error of the mean
SI: Superior-inferior
SP: Senile plaques
St. Louis: Saint Louis
Suppl: Supplementary
TBS: Tris-buffered saline
TBST: Tris-buffer saline containing Tween 20
TC1: Temporal cortex area 1
TF: Time spent with the familiar
TN: Time spent with the novel
TNFα: Tumor necrosis factor-α
TREM2: Triggering receptors expressed on the myeloid cell 2
UK: United Kingdom
USA: United States of America
V: Vehicle
