# Supplementary figures and images for: Clausena Harmandiana root extract attenuated cognitive impairments via reducing amyloid accumulation and neuroinflammation in Aβ1-42-induced rats
Source: BMC Complement Med Ther. 2022 Apr 19;22:108. doi: 10.1186/s12906-022-03591-4 (PMC9019931; doi:10.1186/s12906-022-03591-4)

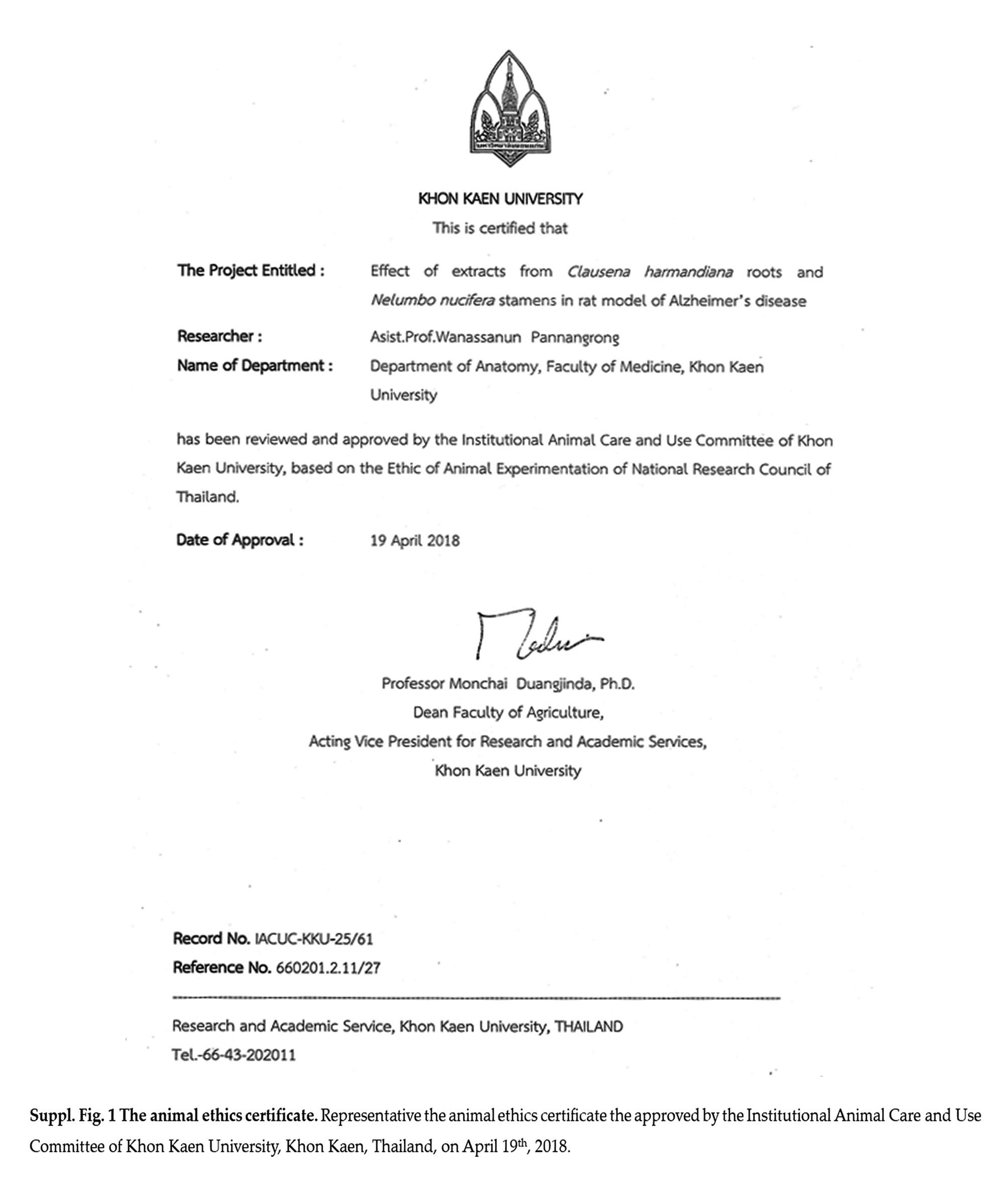

Supplement: Supplementary file 1 — Additional file 1: Suppl. Fig. 1 [file 12906_2022_3591_MOESM1_ESM.tif]

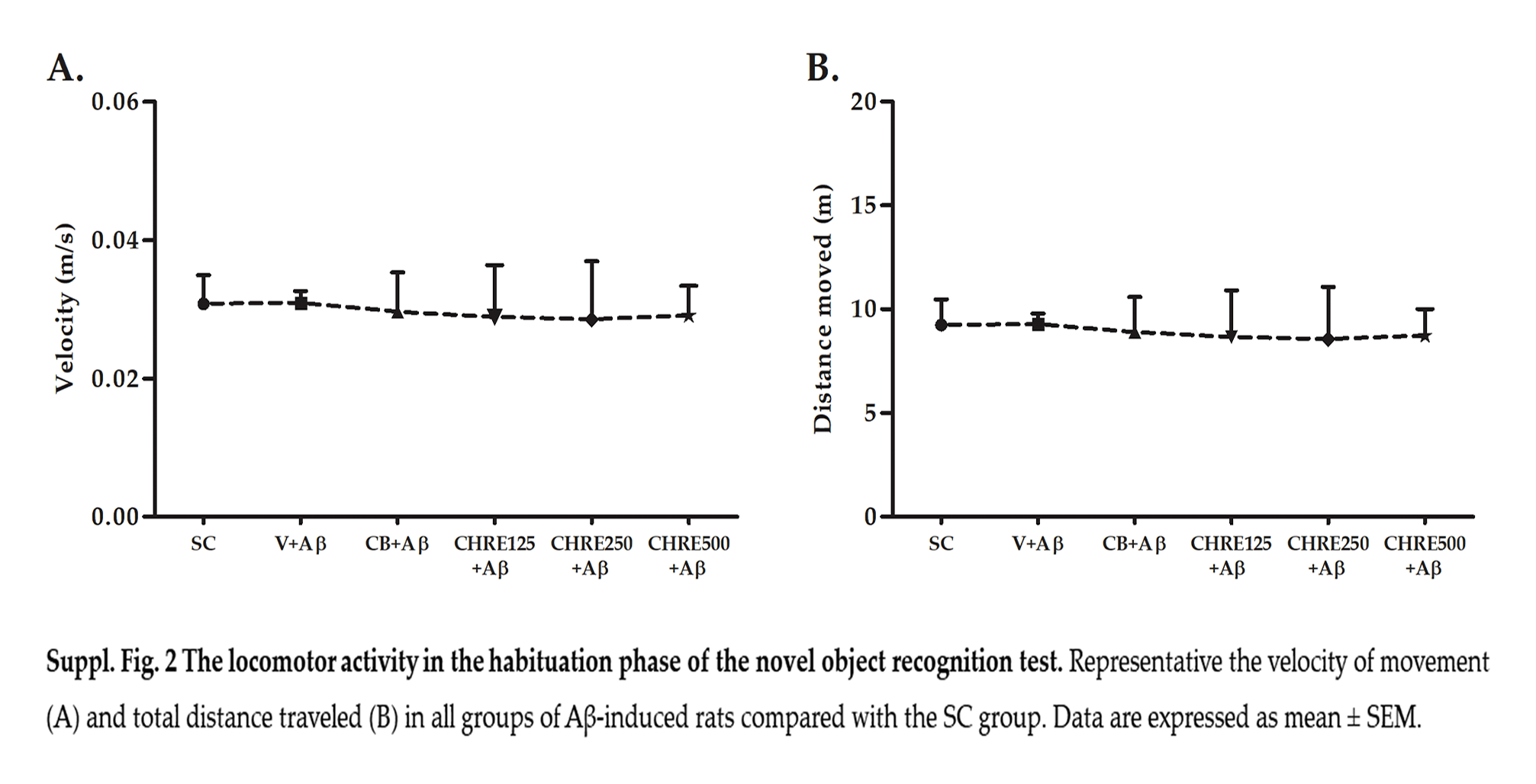

Supplement: Supplementary file 2 — Additional file 2: Suppl. Fig. 2 [file 12906_2022_3591_MOESM2_ESM.tif]

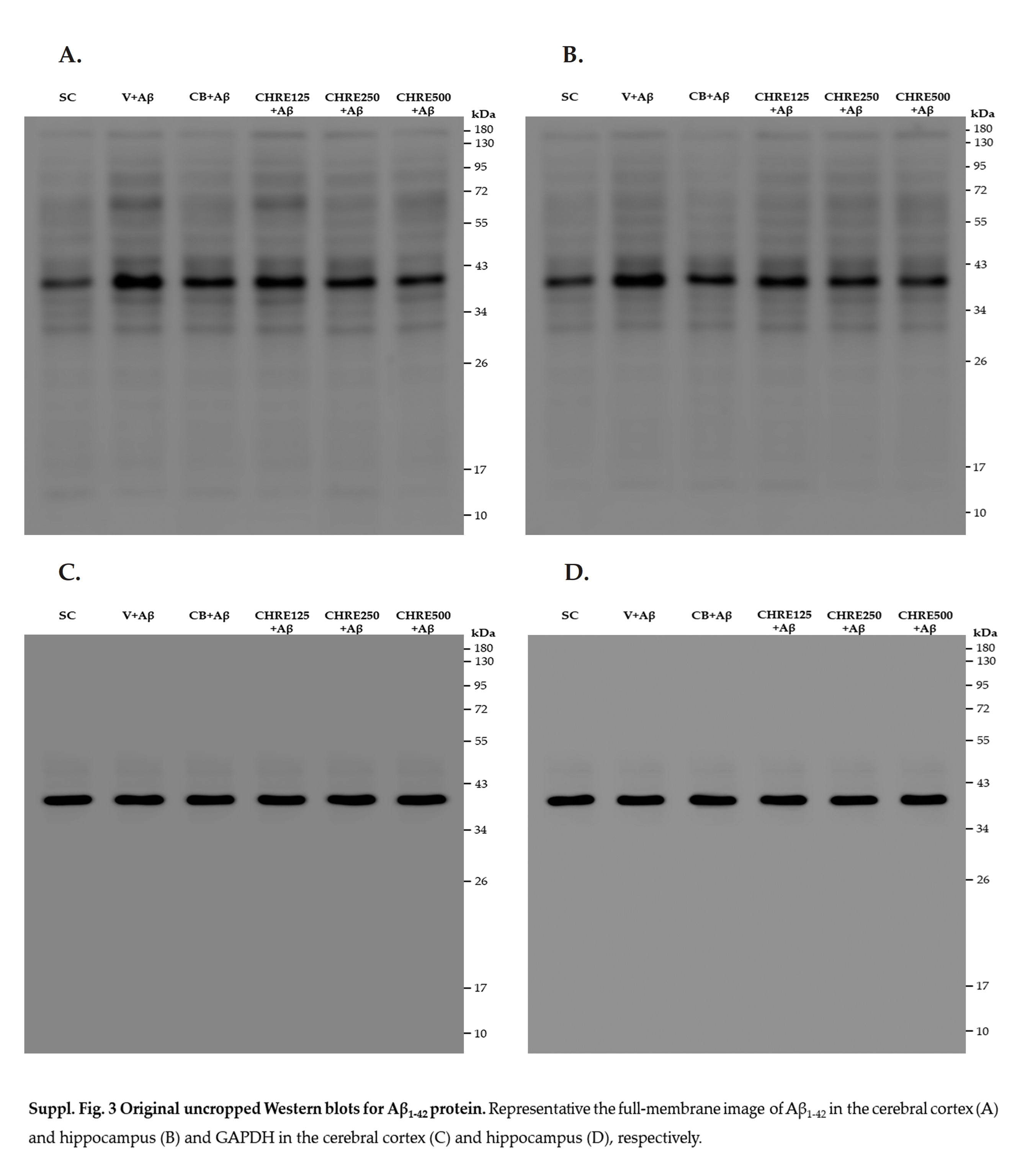

Supplement: Supplementary file 3 — Additional file 3: Suppl. Fig. 3 [file 12906_2022_3591_MOESM3_ESM.tif]

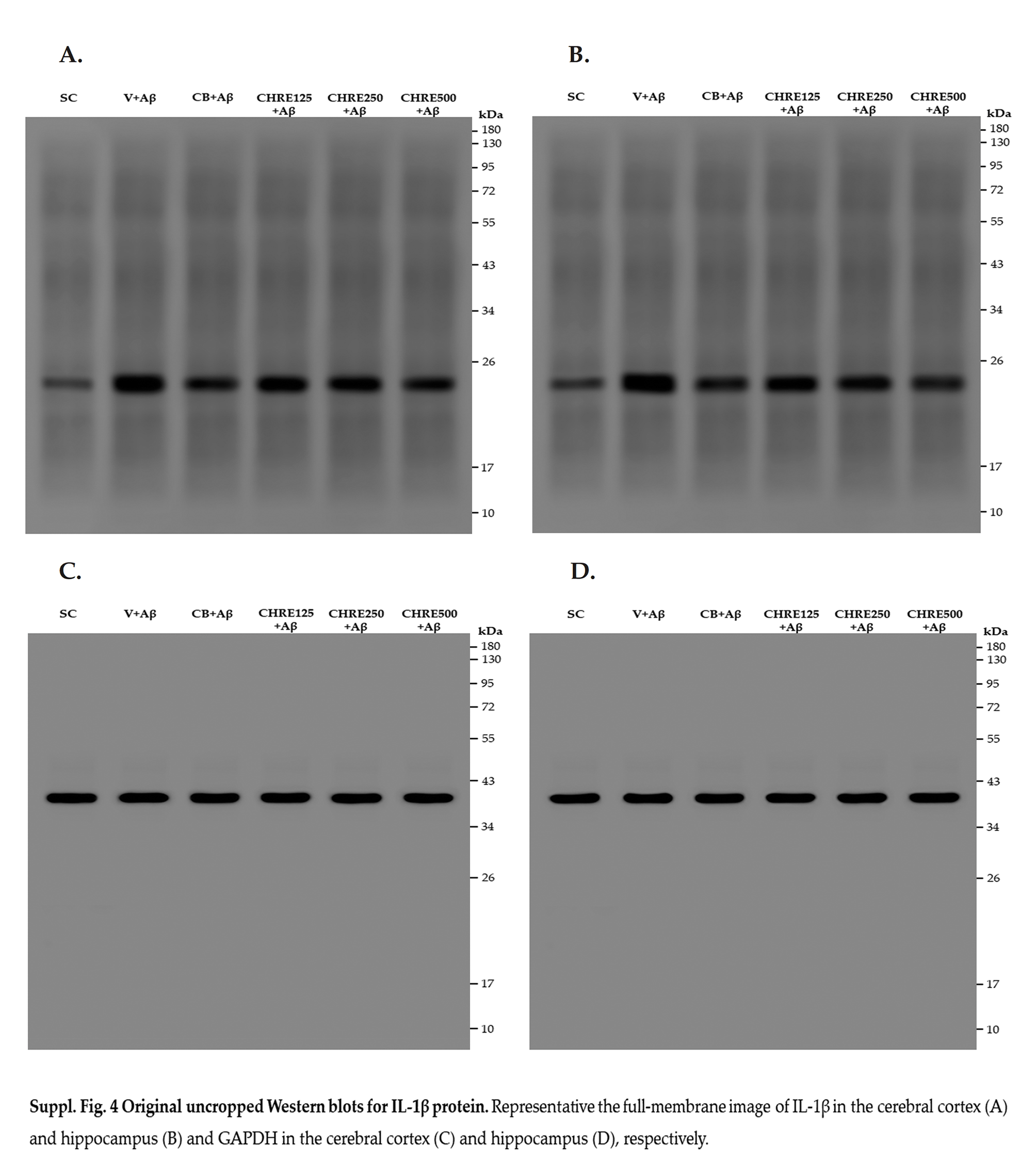

Supplement: Supplementary file 4 — Additional file 4: Suppl. Fig. 4 [file 12906_2022_3591_MOESM4_ESM.tif]

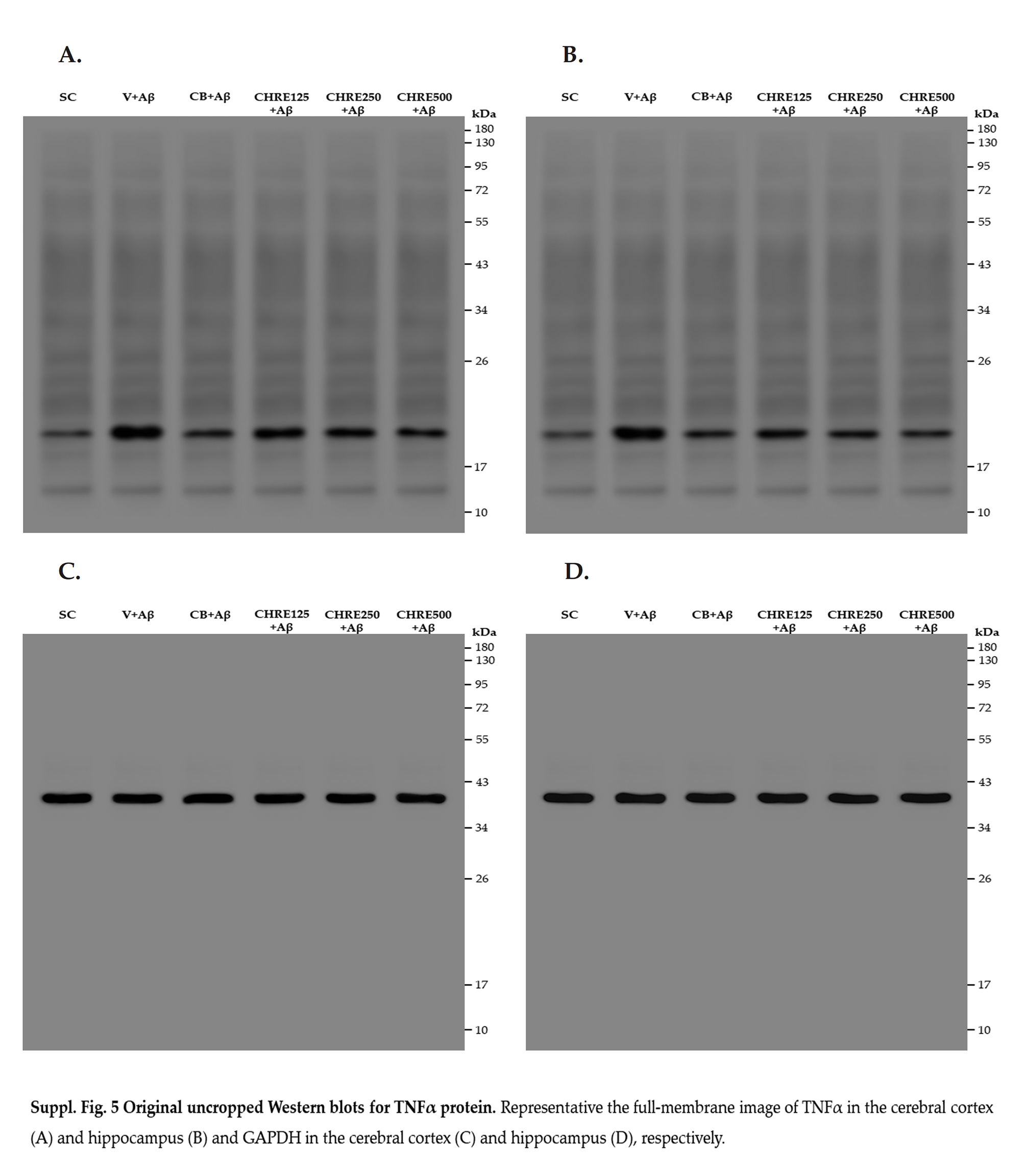

Supplement: Supplementary file 5 — Additional file 5: Suppl. Fig. 5 [file 12906_2022_3591_MOESM5_ESM.tif]
